# miR-7 and miR-153 protect neurons against MPP^+^-induced cell death via upregulation of mTOR pathway

**DOI:** 10.3389/fncel.2014.00182

**Published:** 2014-07-03

**Authors:** Apostolia Fragkouli, Epaminondas Doxakis

**Affiliations:** Lab of Molecular and Cellular Neuroscience, Center for Basic Research, Biomedical Research Foundation of the Academy of AthensAthens, Greece

**Keywords:** Parkinson's disease, miR-7, miR-153, MPP^+^, neuron, neuroprotection, rapamycin, mTOR

## Abstract

Differential expression of microRNAs (miRs) in the brain of patients with neurodegenerative diseases suggests that they may have key regulatory roles in the development of these disorders. Two such miRs, miR-7, and miR-153 have recently been shown to target α-synuclein, a protein critically involved in the pathological process of Parkinson's disease. By using a well-established in culture Parkinson's disease model that of neurotoxin 1-Methyl-4-Phenyl-Pyridinium (MPP^+^), we examined whether miR-7 and miR-153 display neuroprotective properties. Herein, we demonstrate that treatment of cortical neurons with MPP^+^ induced a dose-dependent cell death with apoptotic characteristics. This was reflected in altered intracellular signaling characterized by increased levels of activated kinases p38MAPK and ERK1/2 and reduced levels of activated AKT, p70S6K, and SAPK/JNK. Overexpression of miR-7 or miR-153 by adenoviral transduction protected cortical neurons from MPP^+^-induced toxicity, restored neuronal viability and anti-apoptotic BCL-2 protein levels while attenuated activation of caspase-3. Moreover, both miR-7 and miR-153 interfered with MPP^+^-induced alterations in intracellular signaling pathways in a partially overlapping manner; specifically, they preserved activation of mTOR and SAPK/JNK signaling pathways in the MPP^+^-treated neurons, while miR-153 also attenuated MPP^+^-induced activation of p38MAPK. No major effects were observed in the rest of signaling cascades or proteins investigated. Furthermore, the neuroprotective effect of miR-7 and miR-153 was alleviated when MPP^+^ was co-administered with rapamycin. Taken together, our results suggest that miR-7 and miR-153 protect neurons from cell death by interfering with the MPP^+^-induced downregulation of mTOR signaling.

## Introduction

Parkinson's disease (PD) is a heterogeneous neurodegenerative disorder that affects 1% of the population aged over 65. It perturbs both dopaminergic (substantia nigra pars compacta) and non-dopaminergic (locus coeruleous, raphe nuclei, nucleus basalis of Meynert, hypothalamus, pedunculopontine nucleus) neuronal systems. Our current understanding of the disease points toward a variety of genetic, cellular, and environmental factors that independently or in combination cause progressive neurodegeneration. These factors lead to oxidative stress, abnormal protein degradation, autophagy, reduced protein synthesis, and altered signal transduction that combined induce neuronal death. Which of these mechanisms is more important to PD pathogenesis and progression remains unknown (Obeso et al., 2010). So far, epidemiological data and therapeutic studies using neuroprotective substances such as caffeine, nicotine, ginsenosides, flavonoids, vitamins, and growth factors have pointed out that drugs directed against a single molecular target are likely to be ineffective in treating the disease while agents with multiple pharmacological targets appear more suitable. Consistently, treatments with generic neuroprotective factors and various combinations of approved drugs are now vigorously explored (reviewed in Seidl and Potashkin, 2011; Mythri et al., 2012; Rodnitzky, 2012; Santos, 2012; Kordower and Bjorklund, 2013).

microRNAs (miRs) are a class of highly conserved small, about 22 nucleotides in length, non-coding endogenous RNA molecules that act to inhibit protein expression by partially hybridizing to complementary sequences, in mainly the 3′ UTR, of target RNA transcripts (reviewed in Doxakis, 2013). Each miR is estimated to regulate multiple target mRNAs, and the combinatorial action of miRs is expected to regulate the expression of hundreds of mRNAs. They display a wide variety of expression patterns and many are differentially expressed during development or disease (reviewed in Wienholds and Plasterk, 2005). With respect to PD, it has been shown that two miRs, miR-34, and miR-133, are significantly reduced in affected brain regions relative to controls (reviewed in Mouradian, 2012). Moreover, we and others have reported that two additional miRs, miR-7, and miR-153, target α-synuclein, a protein critically involved in both familial and sporadic pathological processes of PD (Junn et al., 2009; Doxakis, 2010). Importantly, miR-7 and miR-153 are neuron-enriched and show highest levels of expression in murine midbrain (Doxakis, 2010). In addition, miR-7 levels are down-regulated in the midbrain of mice intraperitoneally injected with the PD neurotoxin, MPTP (Junn et al., 2009) while miR-153 has been shown to regulate amyloid β precursor protein (APP) expression and its levels are significantly reduced in Alzheimer's disease brains (Liang et al., 2007; Long et al., 2012). Finally, both miR-7 and miR-153 have been known to modulate intracellular signaling by targeting upstream components of the AKT pathway (Kefas et al., 2008; Fang et al., 2012; Song et al., 2012; Sanchez et al., 2013; Wang et al., 2013; Wu et al., 2013).

1-methyl-4-phenyl-1,2,3,6-tetrahydropyridine (MPTP) is a neurotoxin that was discovered accidentally in exposed humans. Young drug addicts developed an idiopathic parkinsonian syndrome after intravenous self-administration of a synthetic heroin with this contaminant (Davis et al., 1979; Langston et al., 1983). Significantly, most of the biochemical, neuropathological, and clinical characteristics observed, corresponded to the cardinal symptoms of human PD with the exemption of the formation of Lewy bodies (Langston et al., 1983; Ballard et al., 1985). At the molecular level, MPTP is transformed into its toxic derivative 1-methyl-4-phenylpyridinium ion (MPP^+^) by the enzyme MAO-B in astrocytes (Langston et al., 1984; Nicklas et al., 1985). Today, MPTP and MPP^+^ represent the most relevant and frequently used parkinsonian toxins for animal and *in culture* PD models, respectively. A number of studies have, thus far, indicated that inhibition of complex I of the mitochondria electron transport chain, elevation of oxidative stress, activation of pro-apoptotic ERK-1/2 and p38 MAPK and suppression of pro-survival AKT and mTOR signaling pathways contribute to MPP^+^-induced cell death (Mizuno et al., 1987; Deguil et al., 2007; Karunakaran et al., 2008; Cui et al., 2011).

Based on the above, our current study was undertaken to evaluate the ability of miR-7 and miR-153 to prevent MPP^+^-induced toxicity in neurons and delineate the underlying mechanism. Our results demonstrate that miR-7 and miR-153 could protect cortical neurons against MPP^+^-induced death by preserving the activation of the downstream master integrating signaling pathway of mTOR. We argue that these findings may have important therapeutic preclinical applications for PD.

## Materials and methods

### Ethics statement

All rodent tissues were obtained in accordance with European Union (2003/65/CE) guidelines regarding the use of laboratory animals. Experimental protocols were approved by the Institutional Animal Care and Use Committee of BRFAA and the Veterinary Services of Attica prefecture (K/2134).

### Antibodies

The rabbit polyclonal antibodies phospho-S6 ribosomal protein (Ser240/244 CST#2215), phospho-eEF2k (Ser366, CST#3691), phospho-p70 S6 kinase (Thr389, CST#9234), phospho-AKT (Ser473, CST#9271), phospho-ERK1/2 (Thr202/Tyr204, CST#9101), phospho-p38 (Thr180/Tyr182, CST#4511), phospho-SAPK/JNK (Thr183/Tyr185, CST#4668), phospho-GSK3β (Ser9, CST#), phospho-Mapkapk2 (Thr334, CST#3007), S6 ribosomal protein (CST#2217), p70 S6 kinase (CST#9202), AKT (CST#9272), ERK1/2 (CST#9102), p38 (CST#9212), SAPK/JNK (CST#92588), and cleaved caspase-3 (Asp175, CST#9664) were purchased from Cell Signaling Technologies (Beverly, MA, USA). The mouse monoclonal IgG antibodies against BAX (sc-493) and BCL-2 (sc-7382) were purchased from Santa Cruz Biotechnology (Santa Cruz, CA, USA). The anti-GAPDH (GT239) monoclonal antibody was purchased from Genetex (Irvine, CA, USA). The mouse (CST#7076) and rabbit (CST#7074) HRP-conjugated secondary antibodies were from Cell Signaling Technologies.

### Generation of DNA constructs

The construction of pcDNA6.2-GW/EmGFP- scramble/pri-miR-7/pri-miR-153 and pri-miR-7/153 plasmids has been described previously (Doxakis, 2010). The entry plasmids pENTR/EmGFP- scramble/pri-miR-7/pri-miR-153 and pri-miR-7/153 were constructed by inserting the EmGFP-pri-miR cassettes from the pcDNA6.2-GW/EmGFP-pri-miR plasmids into the XhoI/NotI sites of the pENTR Gateway plasmid (Life Technologies, Carlsbad, CA, USA). Using LR clonase II enzyme (Life Technologies) the EmGFP-pri-miR cassettes were, subsequently, transferred by LR recombination from the pENTR plasmid into the pAd5 destination adenoviral vector (Life Technologies). All pAd5/EmGFP-pri-miR vectors were verified by sequencing before use.

### Adenoviral production

pAd5/EmGFP-pri-miR vectors were digested with the PacI enzyme, to lineralize DNA, before transfecting into HEK293A producer cell line in 12-well plates by using Lipofectamine 2000 according to the manufacturer's instructions (Life Technologies). Two days later, cells were trypsinized and transferred onto 10 cm dishes. Culture media were replaced with fresh every 2–3 days until visible regions of cytopathic effect were observed (typically 5–8 days post-transfection). Adenovirus-containing cells and media were harvested when approximately 50% of cells were detached from dish. Crude lysates were prepared by 3 freeze/thaw cycles followed by centrifugation at 3000 rpm for 15 min. To amplify viral stock, 1% of crude adenoviral stocks were used to infect freshly-plated HEK293A cells. Infections were allowed to proceed until 80–90% of the cells have rounded up and were floating (typically 2 days later). High-titer viral stocks were, once again, prepared by 3 freeze/thaw cycles followed by centrifugation at 3000 rpm for 15 min. Adenoviral titers were determined by standard viral plaque assays. Titers were approximately 5 × 10^8^ infectious units per ml.

### Neuron culture and transduction

Dissociated, embryonic day 16 murine cortical neurons (>95% pure, 9 × 10^5^ cells/ml), were grown in Neurobasal/DMEM 1:1 medium (Life Technologies) with 0.5 × B-27 supplement (Life Technologies), 5% heat-inactivated horse serum and Glutamax (Life Technologies) in poly-L-lysine (SIGMA, St-Louis, USA) coated culture plates in the absence of trophic factors (Doxakis et al., 2004). Neurons were transduced by adenoviruses at multiplicity of infection (MOI) 40 at 6–7 days after plating and lysed 48 h post-transduction.

### Pharmacological treatments

MPP^+^ (SIGMA) and rapamycin (SIGMA) were dissolved at the stock concentration of 100 mM in distilled water and tissue culture grade dimethylsulfoxide (DMSO, Applichem, Darmstadt, Germany), respectively. All experiments were initiated at day 7 or 8 after plating when all of the neurons had developed extensive neurite outgrowths. In the case of transduced neurons all pharmacological treatments were performed 24 h post-transduction. In MPP^+^ experiments, neurons were treated with 5–50 μM MPP^+^ for 24 h before analysis while in mTOR experiments, neurons were treated with 20 or 50 nM rapamycin for either 1 h (for signal transduction analysis) or 24 h (for cell viability analysis). When neuronal cultures were co-treated with MPP^+^ and rapamycin, the later compound was added 1 h earlier.

### Methyl thiazol tetrazolium (MTT) assay

MTT assay, a measure of mitochondrial dehydrogenase activity in live cells, was performed in neurons cultured in 96-well poly-L-lysine coated plates. Once the different treatments have been completed, 10 μl of MTT (Applichem) solution in PBS (5 mg/ml) was added to each well and the plate was placed back to incubator for a further 1.5 h. The MTT formazan precipitants formed by live cells were, subsequently, dissolved in 150 μl DMSO and the absorbance was measured at 570 nm by an ELISA microplate reader (ELx800, Bio-Tek Instruments, Winooski, VT, USA).

### Immunoblotting

Immunoblotting was used to assay the protein levels of various intracellular signaling components in 8–9 days old cultures of cortical neurons transduced and/or treated with pharmacological compounds. Neurons were harvested in a lysis buffer containing 25 mM Tris pH 7.5, 150 mM NaCl, 1 mM EDTA, 1%Triton X-100, phosphatase (PhosSTOP®, Roche Applied Sciences, Penzberg, Bavaria, Germany) and protease (Complete®, Roche Applied Sciences) inhibitor cocktails. Cellular protein content was determined by the Bradford assay (Biorad, Hercules, CA, USA). Equal amounts of cell extracts were supplemented with 6x SDS sample buffer (375 mM Tris pH6.8, 10% SDS, 50% glycerol, 10% β-mercaptoethanol, 0.03% bromophenol blue), boiled for 5 min and subjected to SDS-PAGE under reducing conditions on 10 or 12% polyacrylamide gels, depending on the molecular mass of the proteins under examination. After electrophoresis, the resolved proteins were transferred to Protran® nitrocellulose membrane (Whatman, Kent, UK) by electroblotting. Subsequently membranes were saturated for 1 h at room temperature in 5% non-fat milk/0.1% Tween-20 in TBS and incubated at overnight at 4°C in 5% non-fat milk/TBS containing the primary antibody. All primary antibodies were used at 1:1000 dilution as recommended by vendors. The following day, membranes were washed in TBS, incubated for 1 h at room temperature in 5% non-fat milk/TBS containing the appropriate HRP-conjugated secondary antibody, washed in TBS and finally developed using the Western Lighting Plus ECL reagents (PerkinElmer, Waltham, MA, USA) according to the manufacturer's instructions. To ensure equal loading, following film exposure membranes were washed in 0.1% Tween-20 in TBS (TBST), incubated for 30 min at 50°C in stripping buffer (2% SDS, 0.8% mercaptoethanol, 62.5 mM Tris-HCl pH6.8), extensively washed in TBST and after saturation reprobed with the appropriate primary antibodies. Each sample was tested in duplicate and samples obtained from three or four independent experiments were used for analysis. Densitometric analysis of immunoblotting images was performed using the image analysis software Image J, NIH USA.

### Statistical analysis

Mean values were derived from three to five independent experiments performed in duplicate. The effect of treatment on the different parameters examined was assessed using One-Way ANOVA with treatment as independent factor. Bonferroni *post-hoc* analysis was performed where applicable. Significance was defined as *p* < 0.05. All statistical analyses were performed using the SPSS software (Release 10.0.1, SPSS, Chicago, IL, USA).

## Results

### MPP^+^ treatment induced apoptosis in cortical neurons in a concentration-dependent manner, accompanied by alterations in all major intracellular signaling cascades

**Figure 1:**
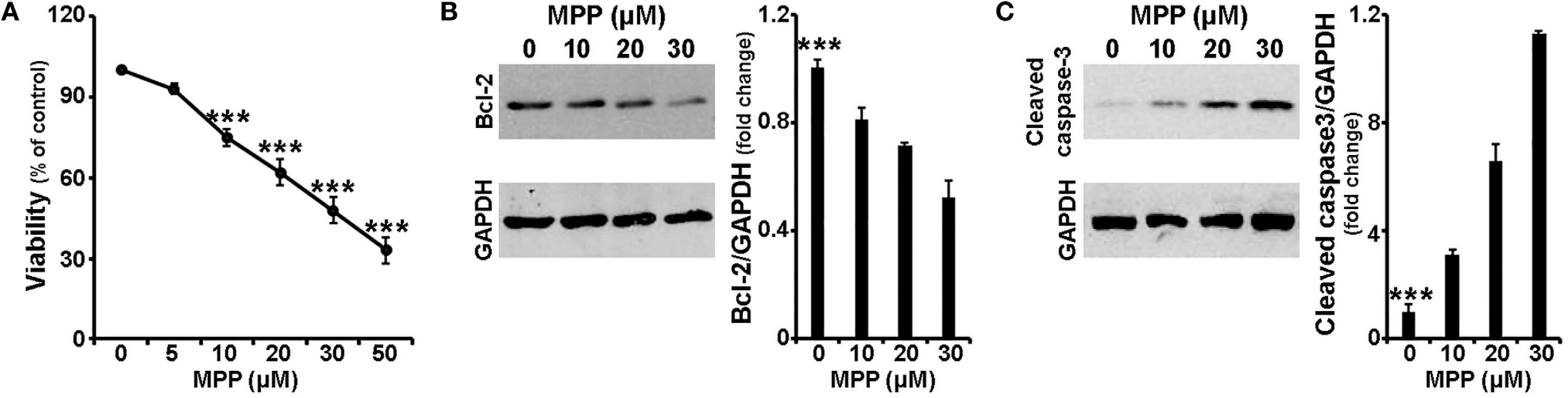
**MPP^+^ induced neurotoxicity in cortical neurons**. Seven-day primary cortical neurons were treated with various concentrations of MPP^+^ for 24 h. **(A)** Cell viability following dose-dependent treatments was assayed by measuring MTT reduction by live neurons. Note that a significant reduction in neuronal viability was observed upon treatment with 10, 20, 30, and 50 μM, but not 5 μM, of MPP^+^. **(B,C)** Equal amounts of total protein from lysates of cortical neurons cultured for 24 h in the presence of 10, 20, and 30 μM MPP^+^ were analyzed on 12% SDS-PAGE and immunoblotted with antibodies specific for BCL-2 **(B)** and cleaved caspase-3 **(C)**. To ensure equal loading, membranes were re-probed against GAPDH. Note that compared to untreated controls, primary cortical neurons treated for 24 h with MPP^+^ displayed a dose-dependent decrease of BCL-2 protein levels, as well as a dose-dependent increase of cleaved caspase-3 protein levels. Quantification of the results in **(B,C)** was performed by scanning densitometry. Bars in all the presented graphs depict mean ± s.e.m. ^***^P ≤ 0.001.

In order to study the mechanism of MPP^+^-induced cell death in our experimental system, 7 days old primary cultures of cortical neurons were treated with various concentrations of MPP^+^ for 24 h. Neuronal viability was, initially, monitored using the MTT assay. As shown in Figure 1A, a 24-h treatment with MPP^+^ induced cytotoxicity in a concentration-dependent manner; loss of viability extended from 7 to 67% with MPP^+^ concentrations ranging between 5 and 50 μM. Statistical analysis revealed a significant effect of MPP^+^ treatment [*F*_(5, 35)_ = 45.662, *P* < 0.001 and *post-hoc*] at 10, 20, 30, and 50 μM, but not 5 μM, of MPP^+^.

Subsequently, the protein levels of the apoptotic-related factors BCL-2, BAX and cleaved caspase-3 were assayed by immunoblot analysis. Consistent with the loss in cell viability, levels of BCL-2, a major pro-survival protein, were significantly reduced by 20, 30, and 48% in cortical neurons treated for 24 h with 10, 20, and 30 μM of MPP^+^, respectively [*F*_(3, 11)_ = 23.699, *P* < 0.001 and *post-hoc*; Figure 1B]. In contrast, a dose-dependent increase was observed in protein levels of cleaved caspase-3, an important effector caspase. More specifically, compared to untreated controls, primary cortical neurons treated for 24 h with 10, 20 and 30 μM of MPP^+^ displayed a significant 3.2-, 6.5-, and 11.3-fold increase of cleaved caspase-3 levels, respectively [*F*_(3, 11)_ = 52.150, *P* < 0.001 and *post-hoc*; Figure 1C]. In our experimental system, the levels of BAX, a major pro-apoptotic factor, were not significantly altered (data not shown). Collectively, these data indicate that MPP^+^ induced a dose-dependent neuronal death that displayed apoptotic features.

**Figure 2:**
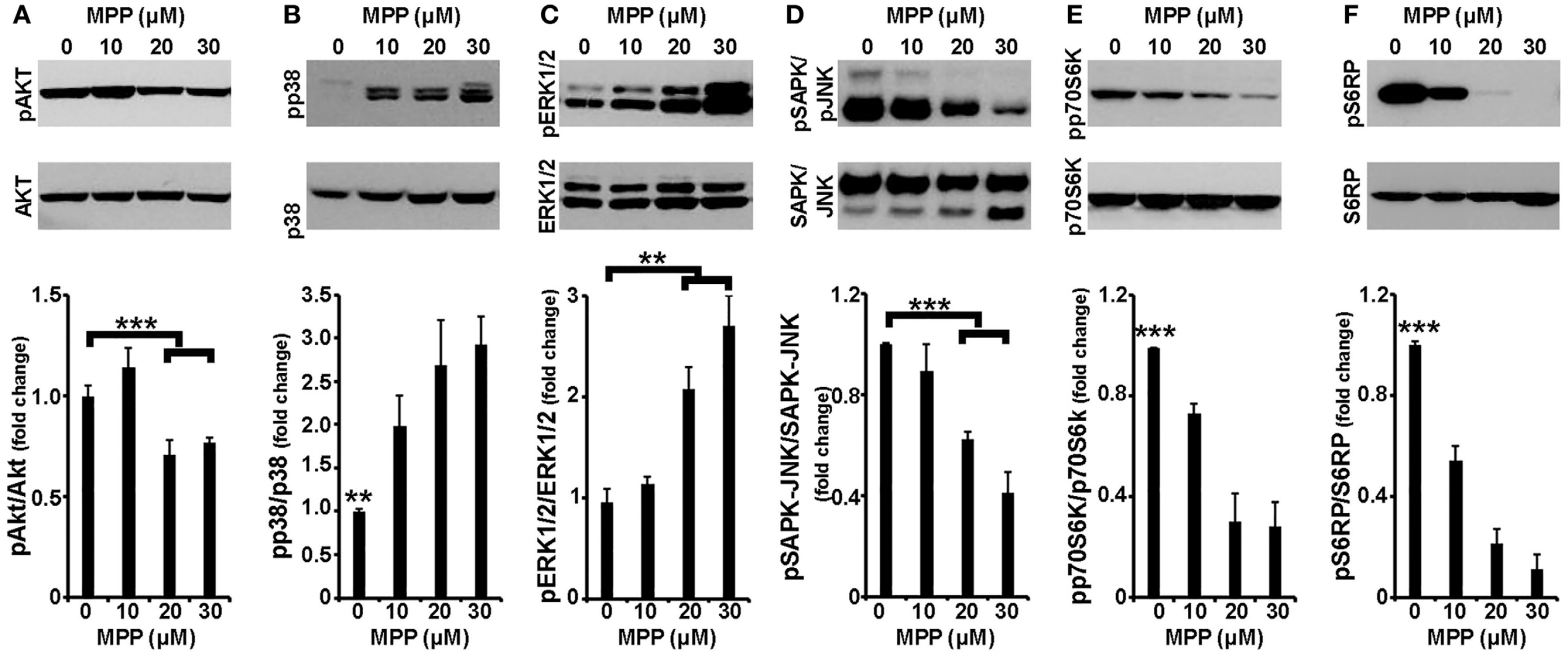
**Cortical neurons treated with MPP^+^ displayed alterations in all major intracellular signaling cascades**. Seven-day primary cortical neurons were treated with 10, 20, and 30 μM MPP^+^ for 24 h. Equal amounts of total protein from lysates of cortical neurons were analyzed on 10% SDS-PAGE and immunoblotted with antibodies specific for phosphorylated forms of AKT **(A)**, p38 MAPK **(B)**, ERK1/2 **(C)**, JNK/SAPK **(D)**, p70S6K **(E)**, as well as for the phosphorylated forms of p70S6K substrate S6RP **(F)**. To ensure equal loading membranes were re-probed against AKT, p38 MAPK, ERK1/2, JNK/SAPK, p70S6K, and S6RP, respectively. Quantification of the results was performed by scanning densitometry. Bars in the graph depict mean ± s.e.m. Note that MPP^+^-treatment changed the phosphorylation status of all the major signaling kinases examined in a concentration dependent manner. ^**^P ≤ 0.01, ^***^P ≤ 0.001.

It is well established that cell apoptosis and survival are regulated by intracellular signaling cascades; thus, activation by phosphorylation of the major signaling effectors was next examined in the same experimental system. As shown in Figures 2A,B, and consistent with previous studies (Junyent et al., 2010; Cui et al., 2011; Hashimoto et al., 2012), the levels of phosphorylated AKT, a major pro-survival kinase, were significantly reduced by ~30% in cortical neurons treated with 20 and 30 μM of MPP^+^ [*F*_(3, 15)_ = 10.932, *P* = 0.001 and *post-hoc*], whereas levels of phosphorylated p38 MAPK, a major pro-apoptotic kinase, were significantly increased at all MPP^+^ concentrations applied [*F*_(3, 15)_ = 5.996, *P* = 0.01 and *post-hoc*]. Similar results were observed in the levels of phosphorylated GSK-3β and MAPKAPK-2, downstream effectors of AKT and p38 MAPK, respectively (data not shown). Moreover, cortical neurons treated for 24 h with 20 or 30 μM, but not 10 μM, of MPP^+^ displayed significant alterations in the levels of phosphorylated ERK1/2, a dubious MAPK, as well as of phosphorylated stress-induced kinases SAPK/JNK; levels of phosphorylated ERK1/2 were up-regulated by 110 and 170% [*F*_(3, 11)_ = 11.806, *P* < 0.01 and *post-hoc*; Figure 2C] and those of phosphorylated SAPK/JNK were down-regulated by 38 and 60% [*F*_(3, 15)_ = 33.699, *P* < 0.001 and *post-hoc*; Figure 2D] at 20 and 30 μM of MPP^+^, respectively. Finally, as shown in Figure 2E, in our experimental system MPP^+^ treatment also significantly reduced the phosphorylation status of the mTOR effector p70S6 kinase, even when applied at 10 μM; this reduction extended from 27% at 10 μM to 72% at 30 μM of MPP^+^ [*F*_(3, 15)_ = 20.701, *P* < 0.001 and *post-hoc*]. Similar reductions were also observed in the levels of activated S6RP [*F*_(3, 15)_ = 60.445, *P* < 0.001 and *post-hoc*; Figure 2F] and of activated eEF2K [*F*_(3, 11)_ = 18.310, *P* = 0.001 and *post-hoc*; data not shown], two p70S6K substrates which mediate translation and cell growth (reviewed in Laplante and Sabatini, 2013). It is therefore evident that in cortical neurons, a 24-h treatment with MPP^+^ leads to changes in the phosphorylation status of all major signaling kinases, in a concentration-dependent manner.

### Over-expression of miR-7 and/or miR-153 in cortical neurons attenuated MPP^+^-induced neurotoxicity

**Figure 3:**
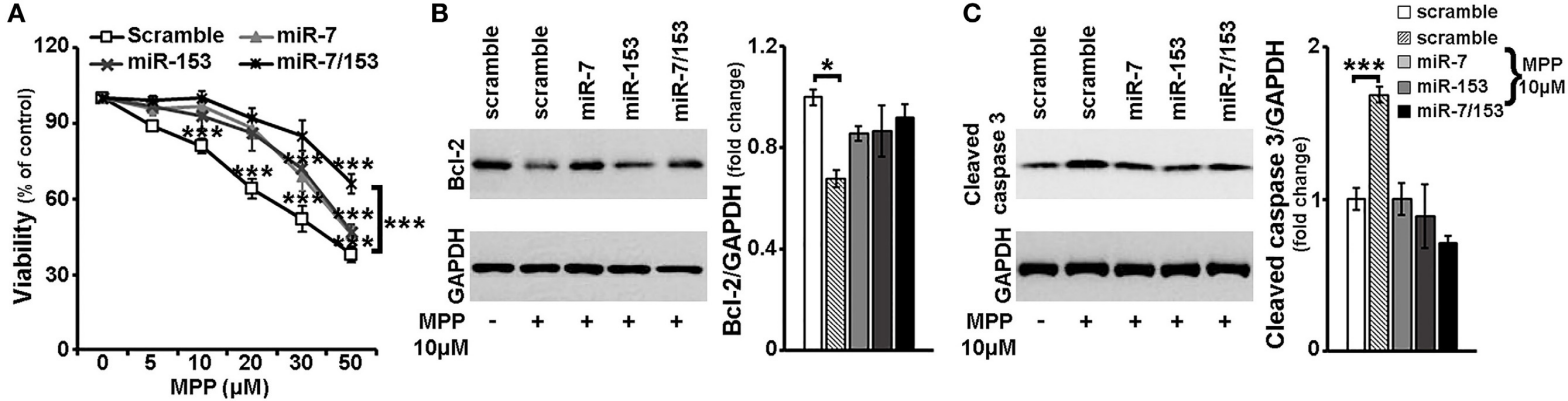
**Neuroprotective effects of miR-7 and miR-153 against MPP^+^-toxicity**. Six to seven days old primary cortical neurons were transduced with adenoviral particles expressing scramble miR, miR-7, miR-153, or both miR-7/153. After 24 h, transduced neurons were exposed for additional 24 h to various concentrations of MPP^+^. **(A)** Neuronal viability following MPP^+^-treatment was monitored by the MTT assay. Note that over-expression of miR-7 and/or miR-153 attenuated MPP^+^-induced cell death. **(B,C)** Equal amounts of total protein from lysates of transduced cortical neurons cultured for 24 h in the presence of 10 μM MPP^+^ were analyzed on 12% SDS-PAGE and immunoblotted with antibodies specific for BCL-2 **(B)** and cleaved caspase-3 **(C)**. To ensure equal loading membranes were re-probed against GAPDH. Quantification of the results was performed by scanning densitometry. Note that compared to scramble-transduced untreated controls, only scramble-transduced cortical neurons displayed significant changes in BCL-2 and cleaved caspase-3 protein levels. Bars in all the depicted graphs correspond to mean ± s.e.m. ^*^P ≤ 0.05, ^***^P ≤ 0.001.

It has been shown that miR-7 and miR-153 target α-synuclein, a protein critically involved in PD pathogenesis (Junn et al., 2009; Doxakis, 2010) and most importantly that miR-7 levels are down-regulated in the midbrain of mice intraperitoneally injected with the PD neurotoxin, MPTP (Junn et al., 2009). Therefore, in order to evaluate possible neuroprotective effects of miR-7 and/or miR-153 against MPP^+^ insult, 6- to 7-day old primary cortical neurons were transduced with adenoviral particles expressing scramble miR, miR-7, miR-153, or both of these two miRs, miR-7/153. It should be noted that irrespective of the adenoviral particles used, overall adenoviral infection of primary cortical neurons affected cell viability, whereas adenoviral over-expression of miR-7 and/or miR-153 had no effect on neuronal viability compared to adenoviral expression of scramble miR (Supplemental Figure 1). Thus, in order to avoid any confounding effects due to the infection *per se*, all subsequent comparisons were performed between primary neurons transduced with adenoviral particles expressing a scramble miR and primary neurons transduced with adenoviral particles expressing the miR(s) of interest. Twenty four hours post-transduction, cortical neurons were exposed to MPP^+^ concentrations ranging between 5 and 50 μM and left *in culture* for additional 24 h. Neuronal viability was then monitored by the MTT assay. As shown in Figure 3A and similar to uninfected neuronal cultures (see Supplemental Figure 2) in the scramble miR transduced cultures loss of viability extended from 10 to 62% with MPP^+^ concentrations ranging between 5 and 50 μM. Statistical analysis revealed a significant effect of MPP^+^ treatment [*F*_(5, 29)_ = 48.968, *P* < 0.001 and *post-hoc*] at 10, 20, 30, and 50 μM, but not 5 μM, of MPP^+^. In contrast, neuronal viability in miR-7 or miR-153 transduced cultures was not impaired when treated with 5, 10, or 20 μM of MPP^+^ and it was only reduced at the higher concentrations applied i.e., 30 and 50 μM [miR-7: *F*_(5, 29)_ = 32.948, *P* < 0.001 and *post-hoc*; miR-153: *F*_(5, 29)_ = 24.816, *P* < 0.001 and *post-hoc*; Figure 3A]. Interestingly, in neuronal cultures transduced with adenoviral particles expressing both miR-7/153 neuronal viability was only impaired upon 24-h treatment with 50 μM MPP^+^ [*F*_(5, 29)_ = 11.803, *P* < 0.001 and *post-hoc*; Figure 3A], Nevertheless, even at the highest concentration applied, compared to scramble miR transduced control cultures, neuronal cultures transduced with both miR-7/153 displayed an approximately 2-fold increase in neuronal viability upon 24-h treatment with 50 μM MPP^+^ [*F*_(3, 19)_ = 11.444, *P* < 0.001 and *post-hoc*; Figure 3A].

Finally, as depicted in Figures 3B,C, compared to scramble miR transduced untreated controls, only scramble-transduced cortical neurons displayed a significant decrease in the levels of anti-apoptotic BCL-2 [*F*_(4, 14)_ = 4.567, *P* < 0.05 and *post-hoc*], accompanied by a significant increase of cleaved caspase-3 levels [*F*_(4, 14)_ = 10.438, *P* = 0.001 and *post-hoc*], upon 24-h treatment with 10 μM of MPP^+^. No such changes were observed among scramble-transduced untreated controls and MPP^+^-treated neuronal cultures transduced with miR-7, miR-153, or miR-7/153 adenoviruses, suggesting that over-expression of miR-7 and miR-153 in cortical neurons attenuated both the MPP^+^-induced down-regulation of pro-survival BCL-2 protein and activation of the pro-apoptotic caspase-3.

### miR-7 and miR-153 activated p70s6K signaling cascade in primary cortical neurons and attenuated the effects of rapamycin on mTOR signaling and cell viability

**Figure 4:**
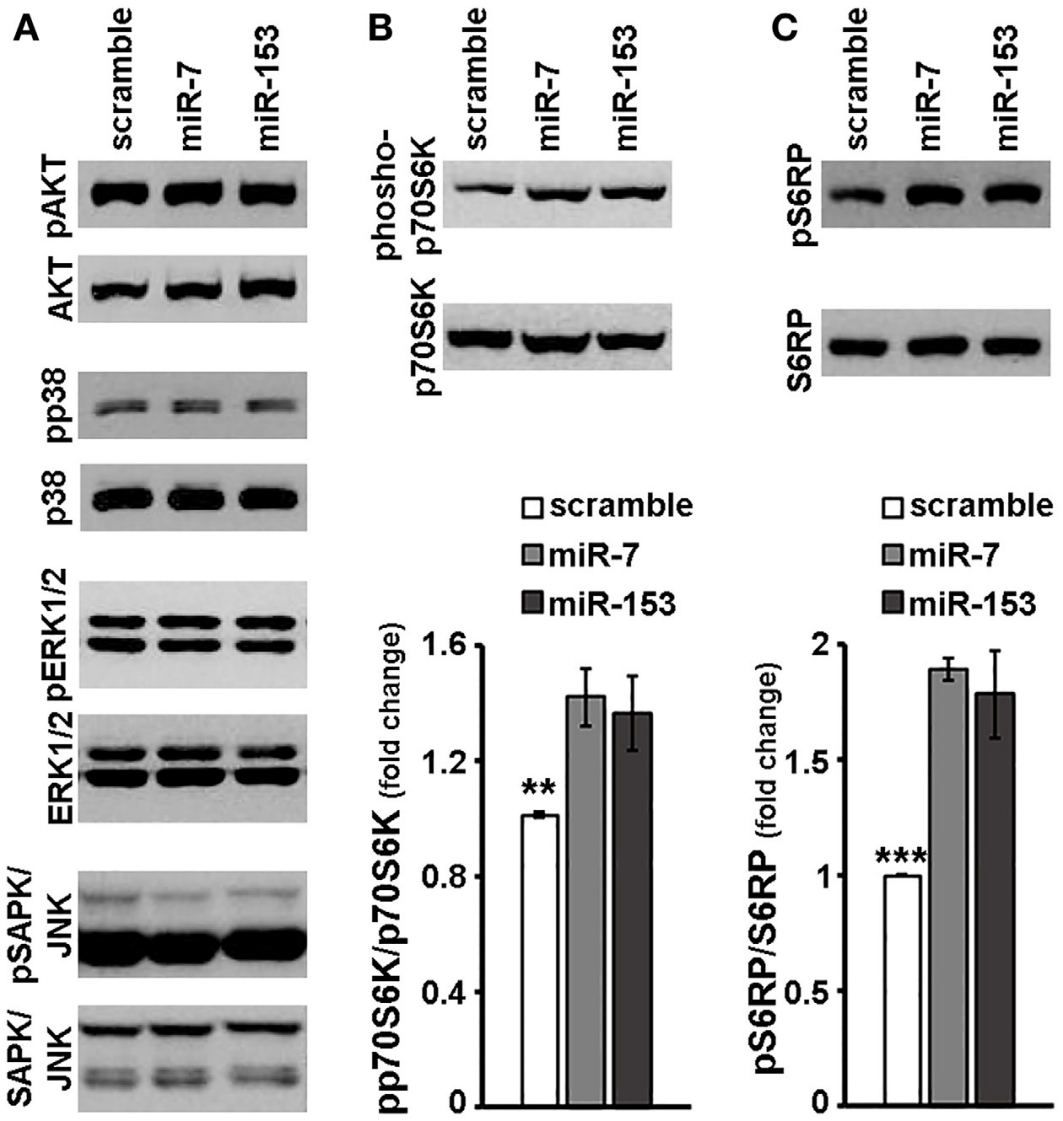
**miR-7 and miR-153 activated p70S6K signaling cascade in cortical neurons**. Six to seven days old primary cortical neurons were transduced with adenoviral particles expressing scramble miR, miR-7, or miR-153 and were lysed 48 h post-transduction. **(A)** Equal amounts of total protein from lysates of cortical neurons were analyzed on 10% SDS-PAGE and immunoblotted with antibodies specific for phosphorylated forms of AKT, p38 MAPK, ERK1/2 SAPK/JNK. To ensure equal loading membranes were re-probed against AKT, p38 MAPK, ERK1/2, and SAPK/JNK, respectively. **(B,C)** Equal amounts of total protein from lysates of cortical neurons were analyzed on 10% SDS-PAGE and immunoblotted with antibodies specific for phosphorylated forms of p70S6K **(B)** as well as for the phosphorylated forms of p70S6K substrate, S6RP **(C)**. To ensure equal loading, membranes were re-probed against p70S6K and S6RP, respectively. Quantification of the results was performed by scanning densitometry. Bars in the graph depict mean ± s.e.m. Note that compared to scramble-transduced controls, primary cortical neurons transduced with miR-7 or miR-153 expressing adenoviruses displayed a significant increase only in the levels of phosphorylated forms of p70S6K and S6RP. ^**^P ≤ 0.01, ^***^P ≤ 0.001.

Given a number of studies that show that miR-7 and miR-153 modulate intracellular signaling (Kefas et al., 2008; Fang et al., 2012; Song et al., 2012; Sanchez et al., 2013; Wang et al., 2013, Wu et al., 2013) in non-neuronal cells, we next investigated whether these miRs affect the activation of the major intracellular signaling cascades in neurons. Therefore, 6–7 days old primary cortical neurons were transduced with adenoviral particles expressing scramble miR, miR-7, or miR-153, and the activation of signaling kinases was assessed 48 h later by immunoblotting. As depicted in Figure 4A, overexpression of miR-7 or miR-153 in cortical neurons did not affect the phosphorylation status of pro-survival kinase AKT or of the pro-apoptotic p38 MAPK and similar results were also observed in the levels of phosphorylated GSK-3β and MAPKAPK-2, downstream effectors of AKT and p38 MAPK, respectively (data not shown). Finally, no significant change was either observed in the levels of phosphorylated ERK1/2 or those of activated stress-induced kinases SAPK/JNK (Figure 4A).

Markedly, in the same experimental system, both miR-7 and miR-153 appeared to induce p70S6 kinase signaling downstream of the mTOR signaling cascade. More specifically, levels of phosphorylated p70S6K were significantly up-regulated by 45 and 52% in cortical neurons transduced with adenoviral particles expressing miR-7 and miR-153, respectively [ANOVA: *F*_(2, 14)_ = 8.056, *P* < 0.01 and *post-hoc*; Figure 4B]. Consistent with the above, phosphorylation of p70S6K substrates S6RP and eEF2K was also significantly increased; levels of phosphorylated S6RP were up-regulated by 89 and 83% [ANOVA: *F*_(2, 11)_ = 20.084, *P* < 0.001 and *post-hoc*; Figure 4C], whereas levels of phosphorylated eEF2K were increased by 36 and 62% [ANOVA: *F*_(2, 11)_ = 7.475, *P* < 0.05 and *post-hoc*; data not shown] upon overexpression of miR-7 and miR-153, respectively. The latter mTOR downstream activation by miR-7 or miR-153 is unlikely to be attributed to an unspecific scramble miR effect, since transduction with adenoviral particles expressing scramble miR appeared to have no effect on the phosphorylation of p70S6K and of its substrate S6RP compared to transduced empty control neurons (Supplemental Figure 3). Taken together the latter observations suggest that miR-7 and miR-153 may activate the mTOR signaling cascade.

**Figure 5:**
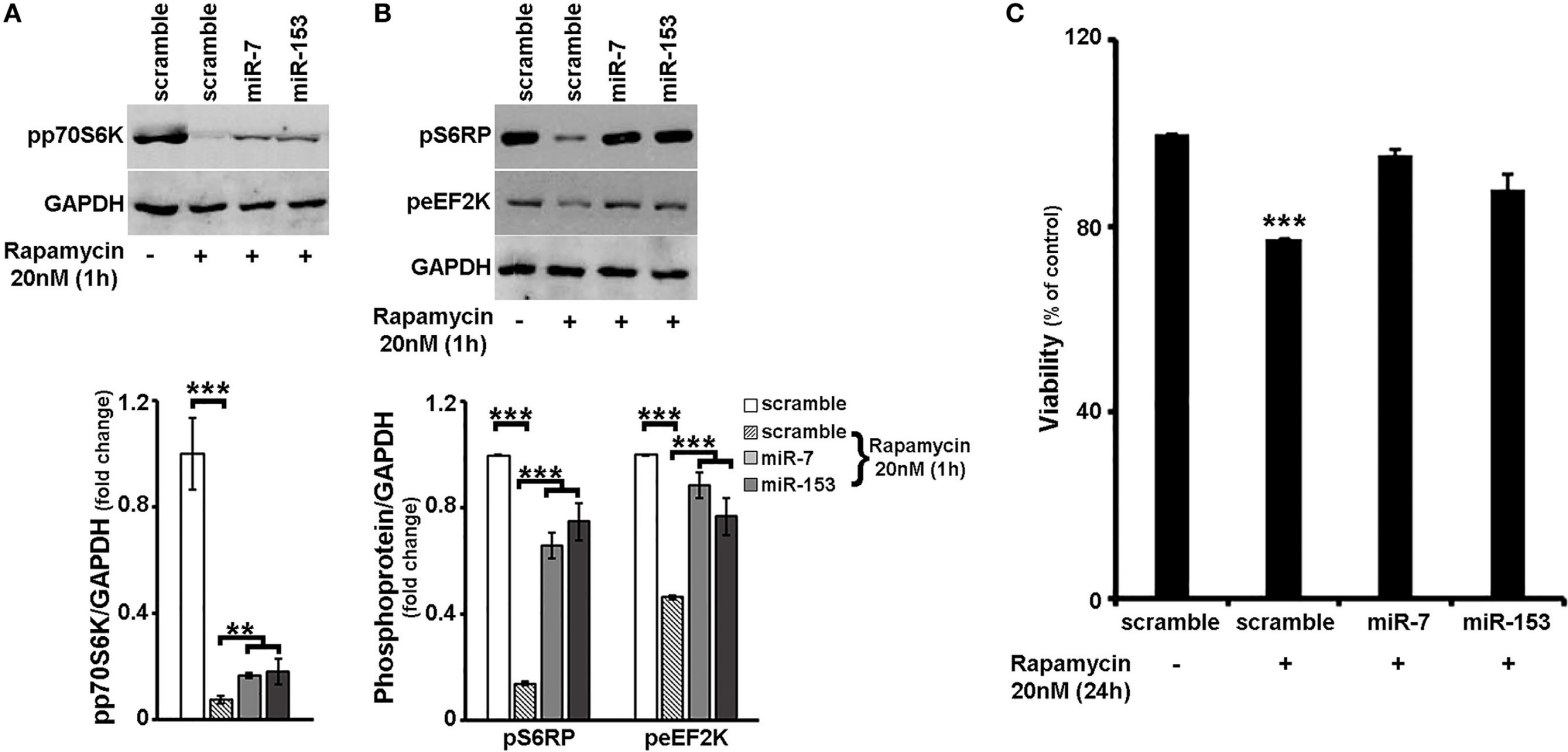
**Overexpression of miR-7 or miR-153 attenuated the effects of rapamycin in cortical neurons. (A,B)** Six to seven days old primary cortical neurons were transduced with adenoviral particles expressing scramble miR, miR-7, or miR-153 and left *in culture* for additional 48 h. One hour before harvest, cultures were supplemented with 20 nM rapamycin. Equal amounts of total protein from lysates of cortical neurons were analyzed on 10% SDS-PAGE and immunoblotted with antibodies specific for phosphorylated forms of p70S6K, S6RP, and eEF2K. To ensure equal loading, membranes were re-probed against GAPDH. Quantification of the results was performed by scanning densitometry. Note that overexpression of miR-7, as well as that of miR-153 attenuated the effect of rapamycin on the phosphorylation of all proteins examined. **(C)** Six to seven days old primary cortical neurons were transduced with the same adenoviral particles and after 24 h were exposed to 20 nM of rapamycin for an additional of 24 h. Neuronal viability following 24-h treatment with rapamycin was monitored by the MTT assay. Note that overexpression of miR-7 and/or miR-153 attenuated rapamycin-induced cell death. Bars in all the presented graphs depict mean ± s.e.m. ^**^P ≤ 0.01, ^***^P ≤ 0.001.

To further explore the latter hypothesis, 6–7 days old primary cortical neurons were again transduced with adenoviral particles expressing scramble miR, miR-7, or miR-153 and left *in culture* for additional 48 h. One hour before harvest, cultures were supplemented with 20 nM rapamycin, a potent mTORC1 (and mTORC2 at higher doses and long-term treatment) inhibitor (Sarbassov et al., 2006; Rosner and Hengstschlager, 2008; Chen et al., 2010) and levels of phosphorylated mTORC1 effector p70S6K and its phosphorylated substrates S6RP and eEF2K were determined. As shown in Figure 5A, irrespective of the adenovirus used, 1-h rapamycin treatment resulted to a significant decrease in the levels of phosphorylated p70S6K [ANOVA: *F*_(3, 11)_ = 36.857, *P* < 0.001 and *post-hoc*]; nevertheless, primary cortical neurons transduced with miR-7 or miR-153 sustained phosphorylated p70S6K levels at more than 2-fold higher than scramble miR-transduced controls (*post-hoc, P* < 0.01). Consistently, overexpression of miR-7 and miR-153 attenuated the effect of rapamycin on the phosphorylation of S6RP [ANOVA: *F*_(3, 11)_ = 71.638, *P* < 0.001 and *post-hoc*; Figure 5B], and of eEF2K [ANOVA: *F*_(3, 11)_ = 65.623, *P* < 0.001 and *post-hoc*; Figure 5B], More specifically, compared to scramble-transduced rapamycin-treated controls, primary cortical neurons transduced with miR-7 or miR-153 and treated with rapamycin displayed significantly increased levels of phosphorylated S6RP (4.7- and 5.4-fold increase in the case of miR-7, miR-153 overexpression, respectively; *post-hoc, P* < 0.001), as well as of phosphorylated eEF2K (by 90% for miR-7 and 65% for miR-153, *post-hoc, P* < 0.001). It appears, therefore, that miR-7 and miR-153 may act as “activators” of mTOR signaling pathway.

Given that mTOR signaling pathway is a downstream regulator of neuronal survival (see also Supplemental Figure 4), we next wanted to examine whether overexpression of miR-7 and/or miR-153 is able to interfere with the effect of mTOR signaling inhibition on cell survival. In order to address this, 6–7 days old primary cortical neurons were transduced with adenoviral particles expressing scramble miR, miR-7, or miR-153, supplemented with 20 nM rapamycin 24 h post-transduction, and left *in culture* for an additional 24 h. Neuronal viability was monitored using the MTT assay. As depicted in Figure 5C, a 24-h treatment with rapamycin reduced significantly the viability of scramble-transduced neuronal cultures to 77% [ANOVA: *F*_(3, 11)_ = 32.804, *P* < 0.001 and *post-hoc*], a reduction that was comparable to the one observed in untransduced cortical neuronal cultures (Supplemental Figure 4). In contrast, viability of miR-7-, or miR-153- transduced neuronal cultures was not significantly impaired, providing further support that these two miRs sustain mTOR signaling in neurons.

### Overexpression of miR-7 and/or miR-153 in cortical neurons attenuated MPP^+^-induced neurotoxicity via upregulation of mTOR pathway

**Figure 6:**
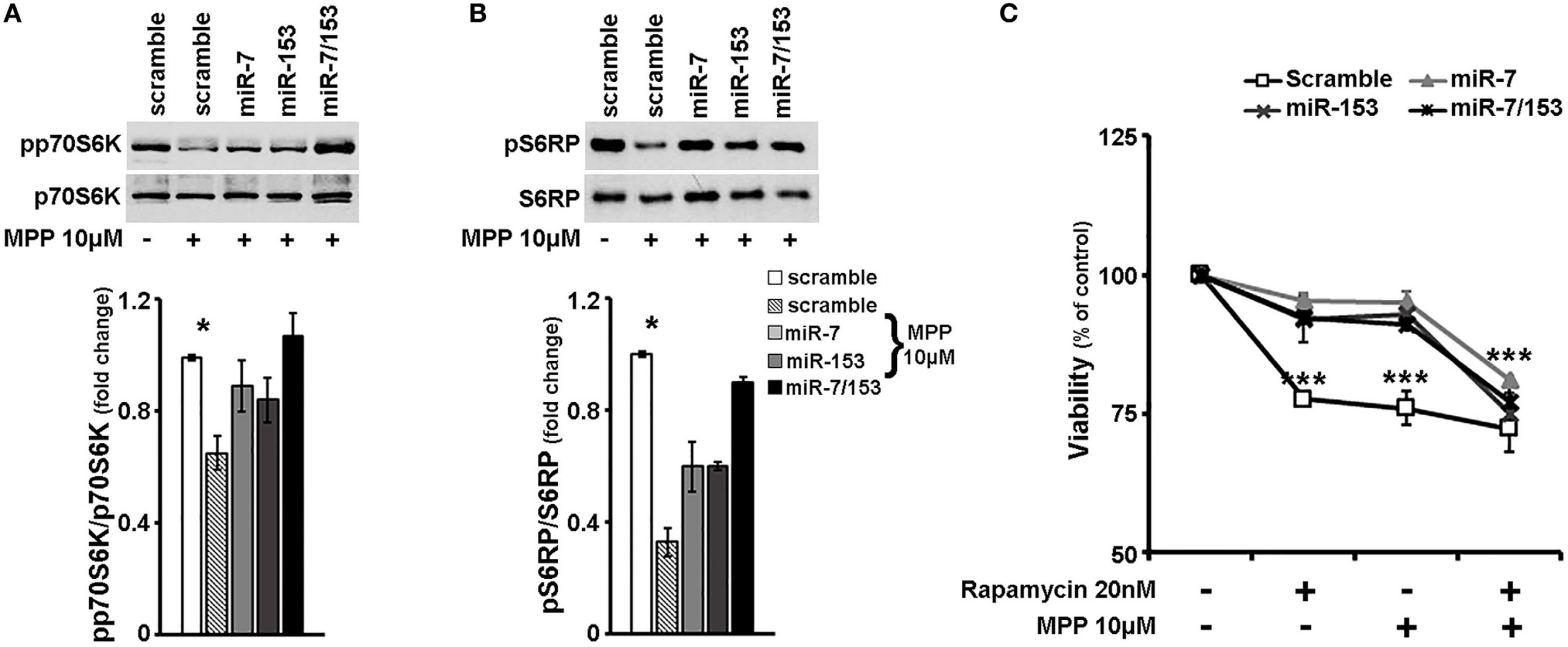
**miR-7 and/or miR-153 attenuated MPP^+^-induced neurotoxicity via upregulation of rapamycin sensitive mTOR pathway. (A,B)** Six to seven days old primary cortical neurons were transduced with adenoviral particles expressing scramble miR, miR-7, miR-153, or both miR-7/153. After 24 h, transduced neurons were exposed for additional 24 h to 10 μM of MPP^+^. Equal amounts of total protein from lysates of transduced cortical neurons cultured for 24 h in the presence of 10 μM MPP^+^ were analyzed on 10% SDS-PAGE and immunoblotted with antibodies specific for phosphorylated forms of p70S6K **(A)** and S6RP **(B)**. To ensure equal loading membranes were re-probed against p70S6K and S6RP, respectively. Note that overexpression of miR-7 and/or miR-153 attenuated the effect of MPP^+^ on the phosphorylation of all proteins examined. Quantification of the results was performed by scanning densitometry. **(C)** Six to seven days old cultures of primary cortical neurons were transduced as described above, and 24 h later were exposed to 20 nM of rapamycin, 10 μM of MPP^+^, or both. Neuronal viability was monitored 24 h post-exposure by the MTT assay. Note that in the presence of rapamycin, overexpression of miR-7 and/or miR-153 failed to protect cortical neurons against MPP^+^-induced cell death. Bars in all presented graphs depict mean ± s.e.m. ^*^P < 0.05, ^***^P ≤ 0.001.

Our results so far suggest that miR-7 and miR-153 are able to induce rapamycin-sensitive mTOR downstream signaling, which appeared significantly impaired in cortical neurons upon MPP^+^ treatment. Therefore, in order to evaluate whether miR-7 and/or miR-153 exert their neuroprotective effect through upregulation of mTOR signaling pathway, 6- to 7- day old primary cortical neurons were again transduced with adenoviral particles expressing scramble miR, miR-7, or miR-153, as well as with an adenoviral construct expressing both of these two miRs. Twenty four hours post-transduction, cortical neurons were exposed to 10 μM of MPP^+^ and the activation of p70S6K and its substrates was assessed 24 h later by immunoblotting. As shown in Figures 6A,B, compared to scramble-transduced untreated controls, 24-h treatment with 10 μM of MPP^+^ induced a significant decrease in the levels of phosphorylated p70S6K [*F*_(4, 14)_ = 5.072, *P* < 0.05 and *post-hoc*] and of its phosphorylated substrate S6RP [*F*_(4, 14)_ = 5.241, *P* < 0.05 and *post-hoc*] in only the scramble-transduced cortical neurons; overexpression of miR-7 or miR-153 attenuated the MPP^+^-induced reduction in the activation of p70S6K and its downstream targets, while overexpression of both miRs restored their phosphorylation status to that of scramble-transduced untreated controls. To further explore the possibility that sustained mTOR downstream signaling activation underlies the neuroprotective effects of miR-7 and miR-153, 20 nM of rapamycin was co-administered with 10 μM MPP^+^ in these neuronal cultures. As shown in Figure 6C, in scramble miR-transduced cultures all treatments lead to a significant reduction in neuronal viability [*F*_(3, 11)_ = 25.244, *P* < 0.001 and *post-hoc*]. In contrast, in miR-7, miR-153, or miR-7/153 -transduced cultures, neuronal viability was significantly impaired only when MPP^+^ was co-administered with rapamycin [miR-7: *F*_(3, 11)_ = 41.253, *P* < 0.001 and *post-hoc*; miR-153: *F*_(3, 11)_ = 14.061, *P* = 0.001 and *post-hoc*, miR-7/153: *F*_(3, 11)_ = 26.653, *P* < 0.001 see Figure 6C]. Taken together the above results suggest that miR-7 and/or miR-153 induced activation of mTOR pathway largely mediates their neuroprotective effect against MPP^+^ toxicity in cortical neurons.

**Figure 7:**
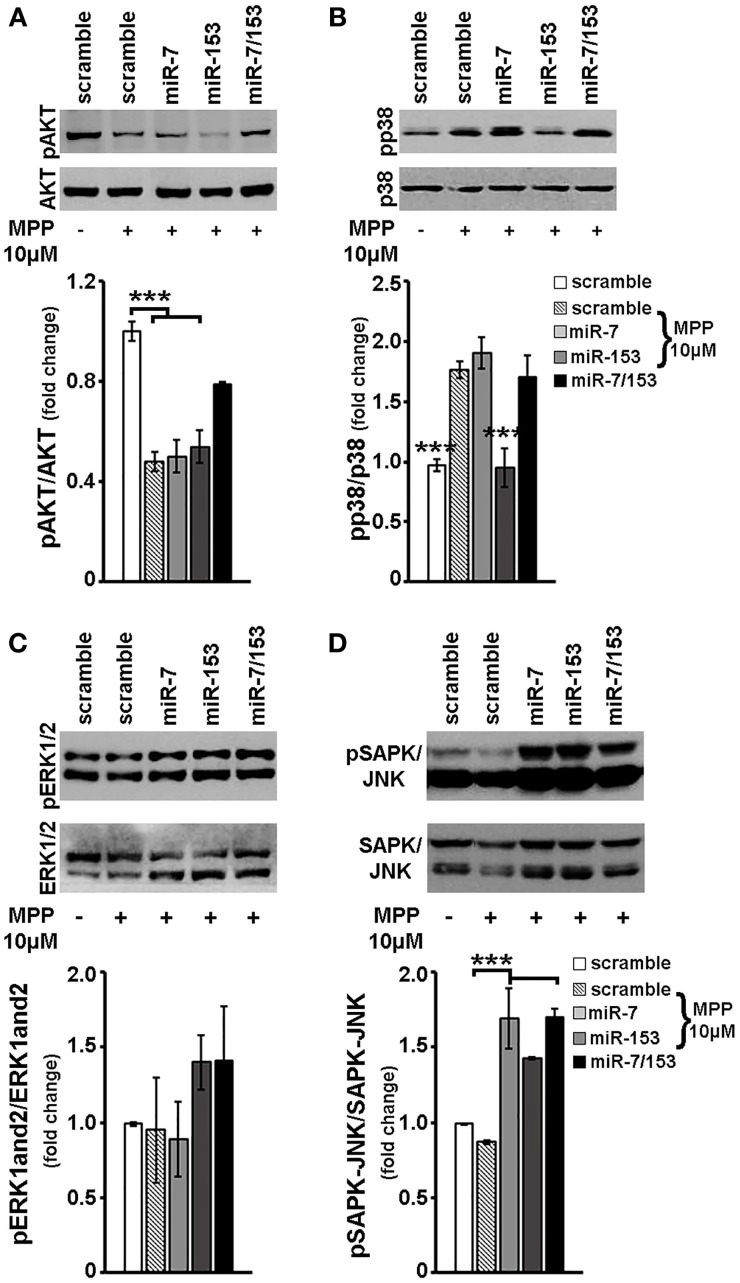
**Effect of miR-7 and/or mir-153 overexpression on AKT, p38, ERK-1/2, and SAPK/JNK signaling in MPP^+^-treated cortical neurons**. Six to seven days old primary cortical neurons were transduced with adenoviral particles expressing scramble miR, miR-7, miR-153, or both miR-7/153. After 24 h, transduced neurons were exposed for additional 24 h to 10 μM of MPP^+^. Equal amounts of total protein from lysates of transduced cortical neurons cultured for 24 h in the presence of 10 μM MPP^+^ were analyzed on 10% SDS-PAGE and immunoblotted with antibodies specific for phosphorylated forms of AKT **(A)**, p38 MAPK **(B)**, ERK1/2 **(C)**, and SAPK/JNK **(D)**. To ensure equal loading membranes were re-probed against AKT, p38 MAPK, ERK1/2, and SAPK/JNK, respectively. Quantification of the results was performed by scanning densitometry. Bars in all the presented graphs depict mean ± s.e.m. Note that miR-7, miR-153, or miR-7/153 altered in an opposite way MPP^+^-induced changes in intracellular signaling cascades. ^***^P < 0.001.

Finally, in order to explore whether miR-7 and/or miR-153 interfere with MPP^+^-induced changes in other than mTOR intracellular signaling cascades, the phosphorylation status of other major signaling effectors was examined in cortical neurons transduced with scramble miR, miR-7, miR-153, or miR-7/153 adenoviruses and treated for 24 h with 10 μM MPP^+^. As shown in Figure 7A, in MPP^+^-treated cortical neurons overexpression of both miR-7/153, but not that of miR-7 or miR-153 alone, attenuated the MPP^+^-induced reduction in the levels of phosphorylated AKT [*F*_(4, 24)_ = 12.314, *P* = 0.01 and *post-hoc*]. Interestingly, miR-153, but not miR-7 or miR-7/153, attenuated the MPP^+^-induced activation of pro-apoptotic p38 MAPK [*F*_(4, 24)_ = 12.978, *P* = 0.01 and *post-hoc*; Figure 7B]. Finally, overexpression of miR-7 and/or miR-153 had no significant effect on the phosphorylation status of ERK1/2 (Figure 7C), but resulted in a significant increase of phosphorylated SAPK/JNK levels to above control levels [*F*_(4, 24)_ = 15.894, *P* < 0.001 and *post-hoc*; Figure 7D]. It, therefore, appears that miR-7 and/or miR-153 alter the intracellular response of cortical neurons to MPP^+^ insult and thus interfere with MPP^+^-induced neurotoxicity.

## Discussion

The mechanisms underlying chronic neurodegeneration in PD remain obscure. An emerging hypothesis is that neuronal systems deteriorate and eventually degenerate due to failure of intrinsic cellular pathways that mediate neuronal homeostasis. This failure maybe due to lack of external neurotrophic support or to mutations in intrinsic factors such as the PARK genes that modify intracellular signaling (reviewed in Wang et al., 2012). Thus, far, a great number of studies have indicated that neurotrophic factors or herbal extracts protect neurons from PD insults by enhancing pro-survival and/or decreasing pro-apoptotic signaling pathways (Nakaso et al., 2008; Wang et al., 2010; Cui et al., 2011; Zhang et al., 2011; Bao et al., 2012; Hashimoto et al., 2012). In addition, manipulation of specific intracellular signaling cascades by either overexpressing or inhibiting signaling protein kinases has revealed that they modulate most PD neurotoxin effects (Malagelada et al., 2006; Zhu et al., 2007, 2012; Nakaso et al., 2008; Cui et al., 2011; Bao et al., 2012; Piao et al., 2012). Most importantly, these findings phenocopy data from the analysis of human postmortem PD brains that show decreased phosphorylation of pro-survival and enhanced activation of pro-apoptotic pathways (Zhu et al., 2002, 2003; Malagelada et al., 2006; Timmons et al., 2009; Reinhardt et al., 2013).

Previous work from our group has shown that mir-7 and mir-153 significantly regulate the expression of α-synuclein, a protein encoded by the gene SNCA that belongs to the PARK gene family (Doxakis, 2010). A-synuclein plays a seminal role in neurodegeneration and has been shown, among others, to affect signaling by modulating neurotrophin BDNF expression and AKT activity (Yuan et al., 2010; Chung et al., 2011). Based on the intrinsic property of miRs to regulate the expression of multiple proteins and possibly the activation of signaling cascades, in the present study we wished to investigate if miR-7 and miR-153 protect neurons exposed to PD insults via altering intracellular signaling. Thus, we evaluated whether overexpression of mir-7 and/or mir-153 could prevent MPP^+^-induced toxicity in cortical neurons. Cortical neurons were selected because they are directly affected in PD by showing progressive pathology (Trojanowski et al., 1998; Braak et al., 2006) and they can be isolated in great numbers relative free of glial cells. MPP^+^, on the other hand, is a widely used neurotoxin that reproduces the neuronal dysfunction of PD both *in vivo* and in different cell systems *in vitro*. MPP^+^ enters cells through the dopamine re-uptake system, present in dopaminergic neurons; however, at higher concentrations it can enter all cell types by passive diffusion (Reinhard et al., 1990) and/or by the extraneuronal monoamine transporter (Russ et al., 1996). The mechanism of MPP^+^ toxicity in cells is rather ubiquitous and involves inhibition of the mitochondrial respiratory chain, elevation of oxidative stress and alteration of intracellular signaling. The vulnerability of neurons to MPP^+^ is modified by microglia numbers in the vicinity of neurons, neurotrophic support, glutathione, or superoxide dismutase content (antioxidant capacity), the content of redox active molecules or elements (such as dopamine or iron), the ratio of anti-apoptotic vs. pro-apoptotic BCL-2 family proteins and basal levels of phosphorylated signaling kinases (Lawson et al., 1990; Kim et al., 2000; Zigmond et al., 2002; Wu et al., 2003; Zecca et al., 2004; Willis et al., 2007). Noteworthy, modulation of intracellular signaling pathways has been shown to mediate most of the MPP^+^ effects in neurons indicating that signaling cascades are downstream of MPP^+^ targets and/or can reverse pro-apoptotic effects (Nakaso et al., 2008; Wang et al., 2010; Cui et al., 2011; Bao et al., 2012; Hashimoto et al., 2012; Piao et al., 2012).

Based on the above, we initially characterized the molecular mechanisms underlying MPP^+^-induced neuronal death in cortical neurons since most studies have been carried out in dividing neuroblastoma cells and/or were limited to two or three signaling pathways. Hence, the levels of apoptosis-related BCL-2 family members and the major signaling pathways, AKT, ERK-1/2, p38, SAPK/JNK, and mTOR were determined. Consistent with previous studies, we found that MPP^+^-induced neurotoxicity displayed apoptotic characteristics, as documented by the reduced levels of BCL-2 and the increased levels of cleaved caspase-3, and was accompanied by enhanced activities of the pro-apoptotic p38 and ERK-1/2 MAPK signaling pathways as well as by reduced activation of pro-survival AKT and p70S6K kinases (Deguil et al., 2007; Junyent et al., 2010; Cui et al., 2011; Bao et al., 2012; Hashimoto et al., 2012; Rodriguez-Blanco et al., 2012). Finally, contrary to most other findings (Wang et al., 2010; Zhang et al., 2011; Hashimoto et al., 2012; Rodriguez-Blanco et al., 2012), and with the exception of a single study (Sun and Chang, 2003), activation of the SAPK/JNK kinase was suppressed in a dose-dependent manner by MPP^+^ treatment of cortical neurons.

Subsequently, the effect of miR-7 and miR-153 overexpression in neurons was determined. We found that miR overexpression did not, overall, alter neuronal viability or the activity of AKT, ERK-1/2, p38, and SAPK/JNK signaling pathways. However, a significant upregulation of mTORC1 downstream signaling was observed by the overexpression of both miRs, as evident by the increased levels of phosphorylated p70S6K and its downstream targets S6RP and eEF2K. mTOR complexes (mTORC1/2) serve as central regulators of cell metabolism, growth and survival by integrating intracellular (energy status, oxygen, and amino acids) and extracellular signals (growth factors) (Wu et al., 2004; Takei et al., 2009; reviewed in Swiech et al., 2008; Laplante and Sabatini, 2013). Mutant mTOR embryos lack telencephalon and die by midgestation, an effect that is phenocopied by the mTOR inhibitor, rapamycin, validating the importance of this pathway in brain development (Hentges et al., 2001). In cultured neurons, mTORC1, the best studied mTOR complex, has been shown to regulate soma size, dendrite axonal growth, dendrite development, and regeneration (Campbell and Holt, 2001; Jaworski et al., 2005; Kumar et al., 2005; Tavazoie et al., 2005; Verma et al., 2005; Li et al., 2008; Park et al., 2008). Our finding that the activation of mTOR downstream effectors was significantly increased in cortical neurons over-expressing miR-7 or miR-153, suggests that these two miRs may act as “activators” of mTOR signaling pathway. The latter hypothesis is further supported by our observations showing that overexpression of miR-7 or miR-153 in primary neurons is able to attenuate the effects of rapamycin on both the activation of mTOR downstream effectors and neuronal viability.

Probing the effect of miR-7 and miR-153 overexpression in MPP^+^-treated neurons, we revealed that they could, either alone or together, significantly protect neurons from cell death. We reasoned that this was due to enhanced mTOR signaling as this was the only pathway that was upregulated by the overexpression of both miRs. Consistent with the latter hypothesis, overexpression of miR-7 and/or miR-153 attenuated the MPP^+^-induced reduction on the activation of p70S6K and its downstream targets, whereas treatment of transduced neurons with rapamycin abolished the pro-survival effects of miR-7 and miR-153 upon MPP^+^ exposure.

To further explore the modulation of intracellular signaling by miR-7 and miR-153 overexpression in MPP^+^-treated neurons, the activation of the remaining pathways was also determined. It should be noted that compared to untransduced cortical neurons, transduced primary cortical neurons used in the present study appeared less resistant to MPP^+^-treatment and therefore displayed a more robust intracellular response to the same MPP^+^ concentration i.e., 10 μM; this is likely to be attributed to the adenoviral transduction, given that it comprises an additional, to that of MPP^+^, insult for the cortical neurons. Taking the latter observation into account, herein we found that the activity of AKT which is known to activate mTORC1 by alleviating the inhibition induced by TSC2 and PRAS40 proteins (Dan et al., 2002; Inoki et al., 2002; Manning et al., 2002; Vander Haar et al., 2007; Zhu et al., 2007), was not restored by either miR-7 or miR-153; however, overexpressing both miR-7 and miR-153 significantly relieved the suppression of AKT activation by MPP^+^, likely by having overlapping or additive effects on their targets. In addition, the finding that AKT was activated at Ser473, known to be mediated by mTORC2 complex (Sarbassov et al., 2005), may indicate that mTORC2 signaling is also contributing to the survival of neurons transduced by both miRs. p38 is a stress kinase that has been linked to neuro-inflammation and MPP^+^-mediated apoptosis (Karunakaran et al., 2008; Thomas et al., 2008b). It should be noted that miR-153, but not miR-7, significantly prevented the activation of p38 by MPP^+^ which may have partly contributed to its pro-survival effects in cortical neurons. Overexpressing miR-7 and miR-153 together alleviated the negative effect of miR-153 on p38 phosphorylation indicating that miR-7 targets may block mir-153 responses on p38 signaling pathway activation. The role of ERK-1/2 activation in neuronal survival is context-specific; some reports show positive or negative input on survival after induction by growth factors, glutamate, or okadaic acid (Runden et al., 1998; Bonni et al., 1999; Satoh et al., 2000; Stanciu et al., 2000; Cui et al., 2011) while others implicate it in MPP^+^- and 6-hydroxydopamine- induced mitophagy/autophagy and cell death (Zhu et al., 2007, 2012). In the present study, neither miR-7 nor miR-153 overexpression significantly changed ERK-1/2 phosphorylation in the MPP^+^-treated neurons. SAPK/JNK is a kinase with an indispensable role in microtubule stability in neurons. It stimulates dendrite formation, axodendritic length, axonal regeneration, mediates fast axonal transport, and contributes to the regulation of synaptic plasticity (Bjorkblom et al., 2005; Chen et al., 2005; Zhu et al., 2005; Tararuk et al., 2006; Thomas et al., 2008a; Morfini et al., 2009; Barnat et al., 2010; Podkowa et al., 2010). At the same time it has been linked to stress-induced apoptosis in different pathological conditions as a result of its inhibition of autophagy and the induction of pro-apoptotic BCL-2 family members (Jia et al., 2006; Hubner et al., 2008; Xu et al., 2011). In our cell culture system, miR-7 and miR-153 overexpression significantly lifted SAPK/JNK activation in the MPP^+^-treated neurons. Overexpression of both miRs together did not further induce SAPK/JNK activation indicating that they modulate a similar target group. Additional experiments will be required to determine if the effect of miR-7 and miR-153 overexpression on SAPK/JNK phosphorylation partly negates their neuroprotective responses via mTOR signaling, and/or maintains the axodendritic growth of neurons which is impaired by MPP^+^-induced microtubule dysfunction (Cartelli et al., 2010) and/or negates MPP^+^-induced ERK-1/2-mediated enhanced autophagy in neurons.

Taken together, our data suggest that miR-7 and miR-153 protect neurons against MPP^+^-induced toxicity via upregulation of mTOR downstream targets. In addition, we show that miR-7 and miR-153 modulate the signaling pathways of SAPK/JNK and p38 in MPP^+^-treated cells, however, their effect on neuronal viability maybe less important. Given also our previous study showing that miR-7 and miR-153 regulate α-synuclein expression, it appears these two miRs may prove good therapeutic candidates for the treatment of PD. Evidence from successful medical interventions based on miRs has already been shown in cornerstone studies to lower plasma cholesterol levels in rodents and primates (Krutzfeldt et al., 2005; Elmen et al., 2008a,b). Currently, a large number of miRs are studied in preclinical and clinical settings by biotechnology companies (Lindow and Kauppinen, 2012). In future, it will be important to characterize the effect of miR-7 and miR-153 on neurite outgrowth and synaptogenesis and test if they can support neurons treated with other PD neurotoxins.

### Conflict of interest statement

The authors declare that the research was conducted in the absence of any commercial or financial relationships that could be construed as a potential conflict of interest.

## Supplementary material

Supplemental Figure 1 **Overexpression of miR-7 or miR-153 has no effect on neuronal viability. (A)** Six to seven days old primary cortical neurons were left untreated or were transduced with adenoviral particles expressing scramble miR, miR-7, miR-153, or both miR-7153 and neuronal viability was assayed by MTT reduction 48 h later. It should be noted that overall adenoviral infection of primary cortical neurons affected cell viability [*F*_(4, 19)_ = 2.979, ^*^*P* = 0.05 and *post-hoc*]; nevertheless, no significant difference was observed among transduced primary neurons, i.e., those transduced with adenoviral particles expressing a scramble miR or the miR (s) of interest. **(B)** Six to seven days old primary cortical neurons were left untreated or were transduced with the same adenoviral particles and lysed 48 h later. Equal amounts of total protein from lysates of cortical neurons were analyzed on 12% SDS-PAGE and immunoblotted with antibodies specific for BCL-2 and cleaved caspase-3. To ensure equal loading membranes were re-probed against GAPDH. Compared to scramble-transduced controls, primary cortical neurons transduced with miR-7, miR-153, or miR-7/153 displayed no significant change on either BCL-2 or cleaved caspase-3 protein levels; it should be noted that a statistically insignificant increase in the levels of cleaved caspase-3 was observed among transduced and untransduced primary neurons.

Supplemental Figure 2 **MMP^+^ treatment induced neurotoxicity and alterations in major signaling cascades in both untransduced and scramble miR transduced cortical neurons in a similar manner. (A)** Six to seven days old primary cortical neurons were left untreated or were transduced with adenoviral particles expressing scramble miR. After 24 h, neuronal cultures were exposed for additional 24 h to various concentrations of MPP^+^. Cell viability following dose-dependent treatments was assayed by measuring MTT reduction by live neurons. Note that similar significant reductions in neuronal viability was observed in both untransduced and scramble miR transduced neurons upon treatment with 10, 20, 30, and 50 μM, but not 5 μM, of MPP^+^. **(B,C)** Six to seven days old primary cortical neurons were left untreated or were transduced with adenoviral particles expressing scramble miR. After 24 h, neuronal cultures were exposed for additional 24 h to 10 μM of MPP^+^. Equal amounts of total protein from lysates of cortical neurons were analyzed on 10% SDS-PAGE and immunoblotted with antibodies specific for phosphorylated forms of AKT, p38 MAPK, p70S6K, SAPK/JNK, and ERK1/2. To ensure equal loading membranes were re-probed against AKT, p38 MAPK, p70S6K, SAPK/JNK, and ERK1/2, respectively. Note that in contrast to untransduced neurons, scramble miR transduced neurons displayed significant reduction of phosphorylated AKT levels even at 10 μM of MPP^+^. No difference was observed in the phosphorylation status of the other major signaling kinases examined among untransduced and scramble miR transduced neurons upon treatment with 10 μM of MPP^+^. ^***^*P* < 0.001.

Supplemental Figure 3 **Adenoviral overexpression of miR-7 and miR-153 in cortical neurons, but not of scramble miR, activated p70S6K signaling cascade**. Six to seven days old primary cortical neurons were transduced with empty adenoviral particles or adenoviral particles expressing scramble miR, miR-7, or miR-153 and were lysed 48 later. Equal amounts of total protein from lysates of untransduced and transduced cortical neurons were analyzed on 10% SDS-PAGE and immunoblotted with antibodies specific for phosphorylated forms of p70S6K **(A)** and S6RP **(B)**. To ensure equal loading, membranes were re-probed against GAPDH. Note that no difference was observed in the levels of phosphorylated p70S6K and S6RP between transduced empty control and scramble miR transduced cortical neurons.

Supplemental Figure 4 **Rapamycin induced neurotoxicity in cortical neurons**. Seven-day primary cortical neurons were treated with various concentrations of rapamycin and MPP^+^ for 24 h. Cell viability following dose-dependent treatments was assayed by measuring MTT reduction by live neurons. Note that a significant ~20% reduction in neuronal viability was observed upon treatment with rapamycin at concentrations 20–50 nM [*F*_(4, 14)_ = 20.852, *P* < 0.001]. No synergistic effect was observed among rapamycin and MPP^+^. ^***^*P* < 0.001.
