# Supplementary figures and images for: miR-7 and miR-153 protect neurons against MPP+-induced cell death via upregulation of mTOR pathway
Source: Front Cell Neurosci. 2014 Jul 3;8:182. doi: 10.3389/fncel.2014.00182 (PMC4080263; doi:10.3389/fncel.2014.00182)

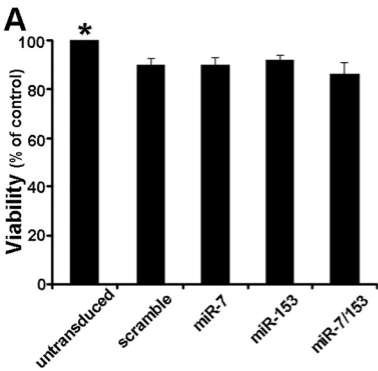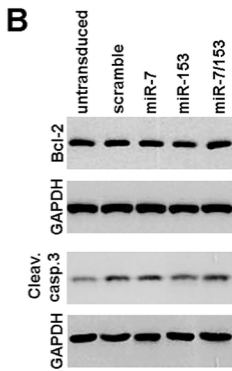

Supplement: Supplemental Figure 1 — Overexpression of miR-7 or miR-153 has no effect on neuronal viability. (A) Six to seven days old primary cortical neurons were left untreated or were transduced with adenoviral particles expressing scramble miR, miR-7, miR-153, or both miR-7153 and neuronal viability was assayed by MTT reduction 48 h later. It should be noted that overall adenoviral infection of primary cortical neurons affected cell viability [F(4, 19) = 2.979, *P = 0.05 and post-hoc]; nevertheless, no significant difference was observed among transduced primary neurons, i.e., those transduced with adenoviral particles expressing a scramble miR or the miR (s) of interest. (B) Six to seven days old primary cortical neurons were left untreated or were transduced with the same adenoviral particles and lysed 48 h later. Equal amounts of total protein from lysates of cortical neurons were analyzed on 12% SDS-PAGE and immunoblotted with antibodies specific for BCL-2 and cleaved caspase-3. To ensure equal loading membranes were re-probed against GAPDH. Compared to scramble-transduced controls, primary cortical neurons transduced with miR-7, miR-153, or miR-7/153 displayed no significant change on either BCL-2 or cleaved caspase-3 protein levels; it should be noted that a statistically insignificant increase in the levels of cleaved caspase-3 was observed among transduced and untransduced primary neurons. [file Presentation1.PDF]

**A**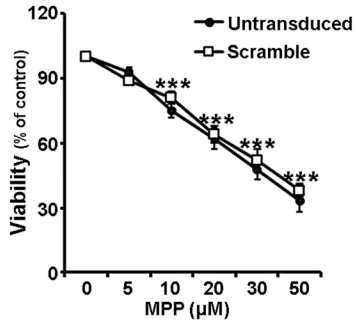**B**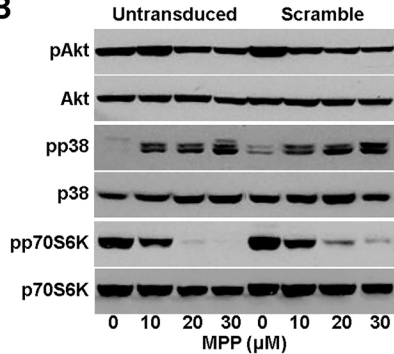**C**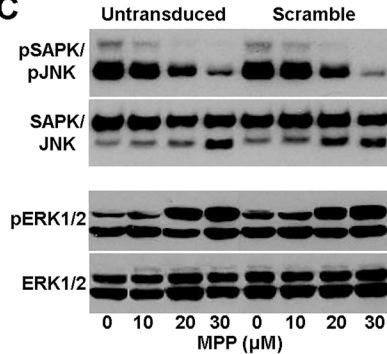

Supplement: Supplemental Figure 2 — MMP+ treatment induced neurotoxicity and alterations in major signaling cascades in both untransduced and scramble miR transduced cortical neurons in a similar manner. (A) Six to seven days old primary cortical neurons were left untreated or were transduced with adenoviral particles expressing scramble miR. After 24 h, neuronal cultures were exposed for additional 24 h to various concentrations of MPP+. Cell viability following dose-dependent treatments was assayed by measuring MTT reduction by live neurons. Note that similar significant reductions in neuronal viability was observed in both untransduced and scramble miR transduced neurons upon treatment with 10, 20, 30, and 50 μM, but not 5 μM, of MPP+. (B,C) Six to seven days old primary cortical neurons were left untreated or were transduced with adenoviral particles expressing scramble miR. After 24 h, neuronal cultures were exposed for additional 24 h to 10 μM of MPP+. Equal amounts of total protein from lysates of cortical neurons were analyzed on 10% SDS-PAGE and immunoblotted with antibodies specific for phosphorylated forms of AKT, p38 MAPK, p70S6K, SAPK/JNK, and ERK1/2. To ensure equal loading membranes were re-probed against AKT, p38 MAPK, p70S6K, SAPK/JNK, and ERK1/2, respectively. Note that in contrast to untransduced neurons, scramble miR transduced neurons displayed significant reduction of phosphorylated AKT levels even at 10 μM of MPP+. No difference was observed in the phosphorylation status of the other major signaling kinases examined among untransduced and scramble miR transduced neurons upon treatment with 10 μM of MPP+. ***P < 0.001. [file Presentation2.PDF]

**A**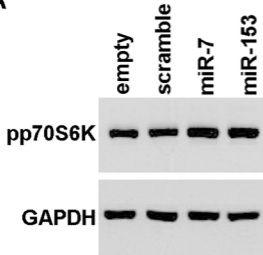**B**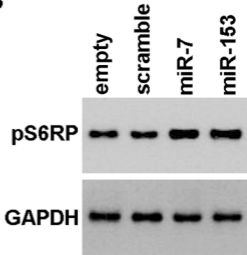

Supplement: Supplemental Figure 3 — Adenoviral overexpression of miR-7 and miR-153 in cortical neurons, but not of scramble miR, activated p70S6K signaling cascade. Six to seven days old primary cortical neurons were transduced with empty adenoviral particles or adenoviral particles expressing scramble miR, miR-7, or miR-153 and were lysed 48 later. Equal amounts of total protein from lysates of untransduced and transduced cortical neurons were analyzed on 10% SDS-PAGE and immunoblotted with antibodies specific for phosphorylated forms of p70S6K (A) and S6RP (B). To ensure equal loading, membranes were re-probed against GAPDH. Note that no difference was observed in the levels of phosphorylated p70S6K and S6RP between transduced empty control and scramble miR transduced cortical neurons. [file Presentation3.PDF]

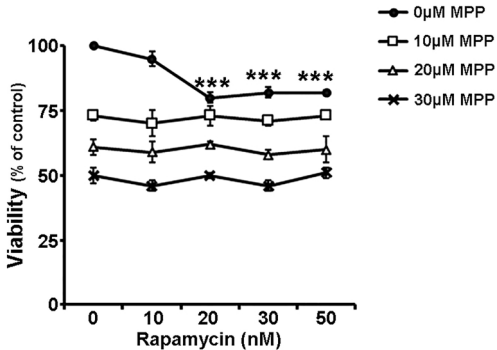

Supplement: Supplemental Figure 4 — Rapamycin induced neurotoxicity in cortical neurons. Seven-day primary cortical neurons were treated with various concentrations of rapamycin and MPP+ for 24 h. Cell viability following dose-dependent treatments was assayed by measuring MTT reduction by live neurons. Note that a significant ~20% reduction in neuronal viability was observed upon treatment with rapamycin at concentrations 20–50 nM [F(4, 14) = 20.852, P < 0.001]. No synergistic effect was observed among rapamycin and MPP+. ***P < 0.001. [file Presentation4.PDF]
